# Supplementary material for: Structural insights into transcription initiation by yeast RNA polymerase I
Source: EMBO J. 2017 Jul 24;36(18):2698–709. doi: 10.15252/embj.201796958 (PMC5599796; doi:10.15252/embj.201796958)
Supplement: Supplementary file 1 — Appendix [file EMBJ-36-2698-s001.pdf]

Appendix

Structural insights into transcription initiation by yeast RNA polymerase I

Yashar Sadian, Lucas Tafur, Jan Kosinski, Arjen J. Jakobi, Rene Wetzel, Katarzyna Buczak, Wim J. H. Hagen, Martin Beck, Carsten Sachse & Christoph W. Müller

Appendix Figure S1
Reconstitution and electron microscopy analysis of the Pol I PIC.

Appendix Figure S2
Cryo-EM processing pipeline.

Appendix Figure S3
Comparison of the crystal structure of CF and the CF model obtained in this work.

Appendix Figure S4
Crystal structure of Core Factor fitted into the Pol I PIC density.

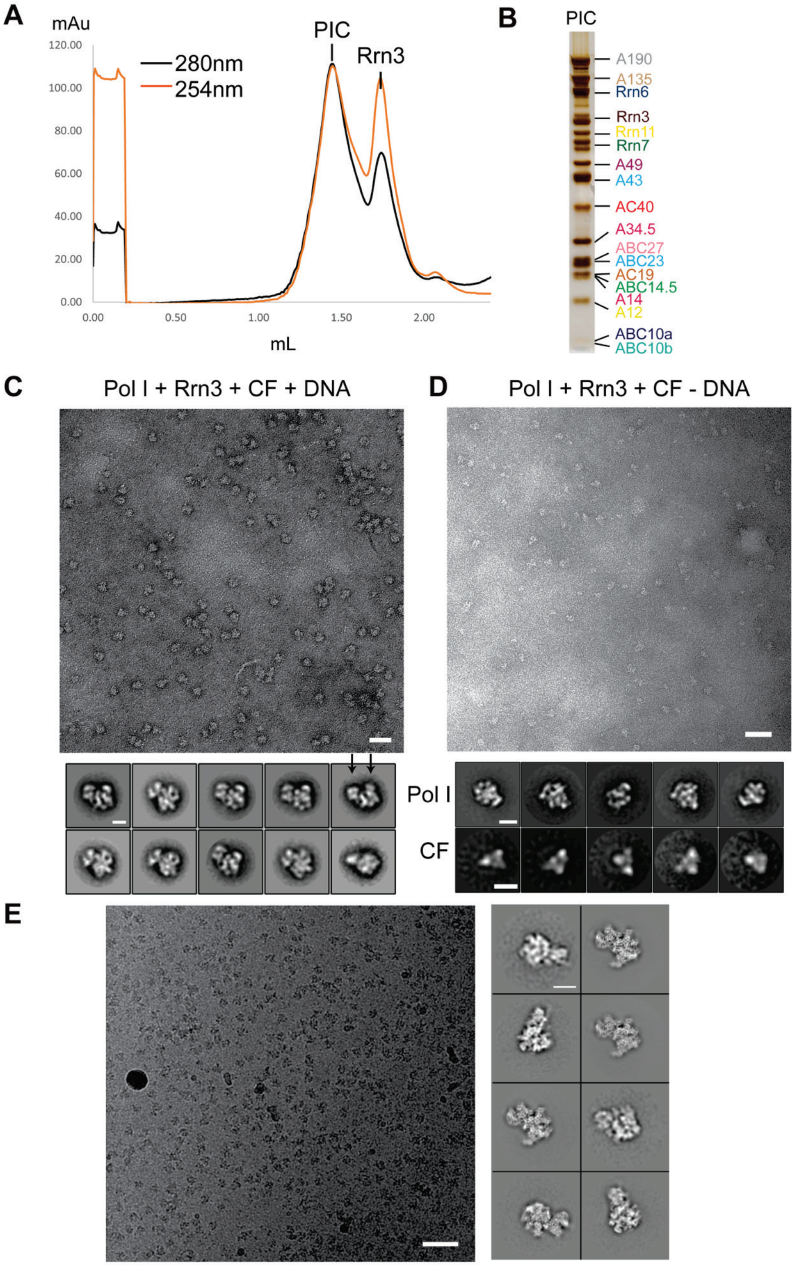

Appendix Figure S1. Reconstitution and electron microscopy analysis of the Pol I PIC. A. Typical gel filtration profile (GE Superose 6 Increase 2.3/300) of the reconstituted Pol 1 PIC showing that the sample is monodisperse. **B.** Denaturing electrophoresis gel stained with silver nitrate shows the presence of all 18 subunits in stoichiometric amounts. **C.** Representative negative stain micrograph and 2D class averages of the sample. The arrows indicate the position of CF and Pol I. **D.** The complex does not form in the absence of DNA. The 2D class averages show the presence of either Pol I or CF, but not the PIC (compare with **C**). **E.** Representative micrograph of the cryo-EM dataset and the class averages obtained from the particles used for final refinement. Scale bars = 50 nm (micrograph) and 10 nm (2D class averages).

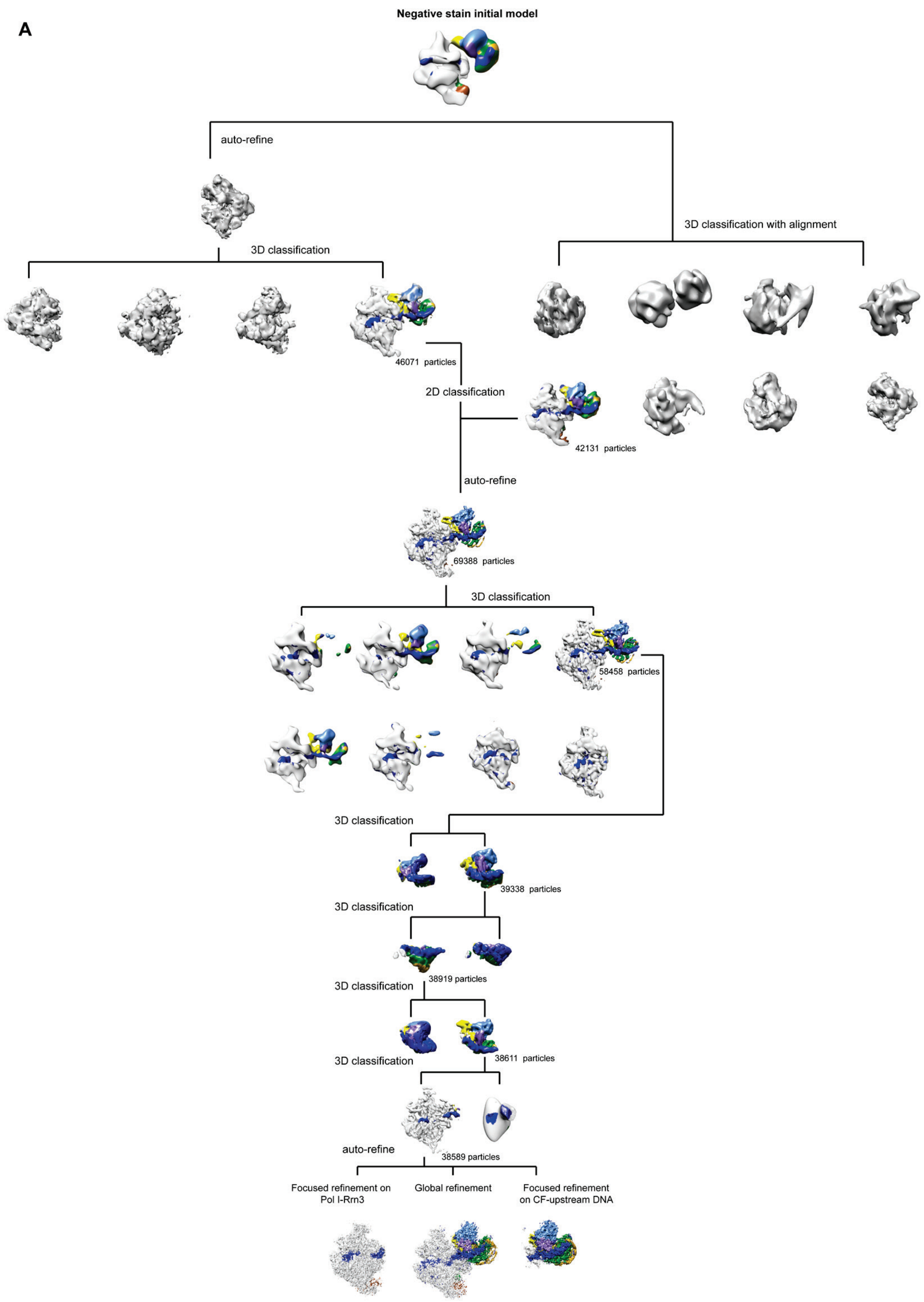

Appendix Figure S2. Cryo-EM processing pipeline. A. Picked particles after 2D classification were sorted extensively by repeated rounds of 3D classification, with and without masking for CF and upstream DNA density (shown colored as in Fig 1). The final particles (bottom) were refined without a mask (global refinement, center) and focusing on either Pol I-Rrn3 (left) or CF-upstream DNA (right).

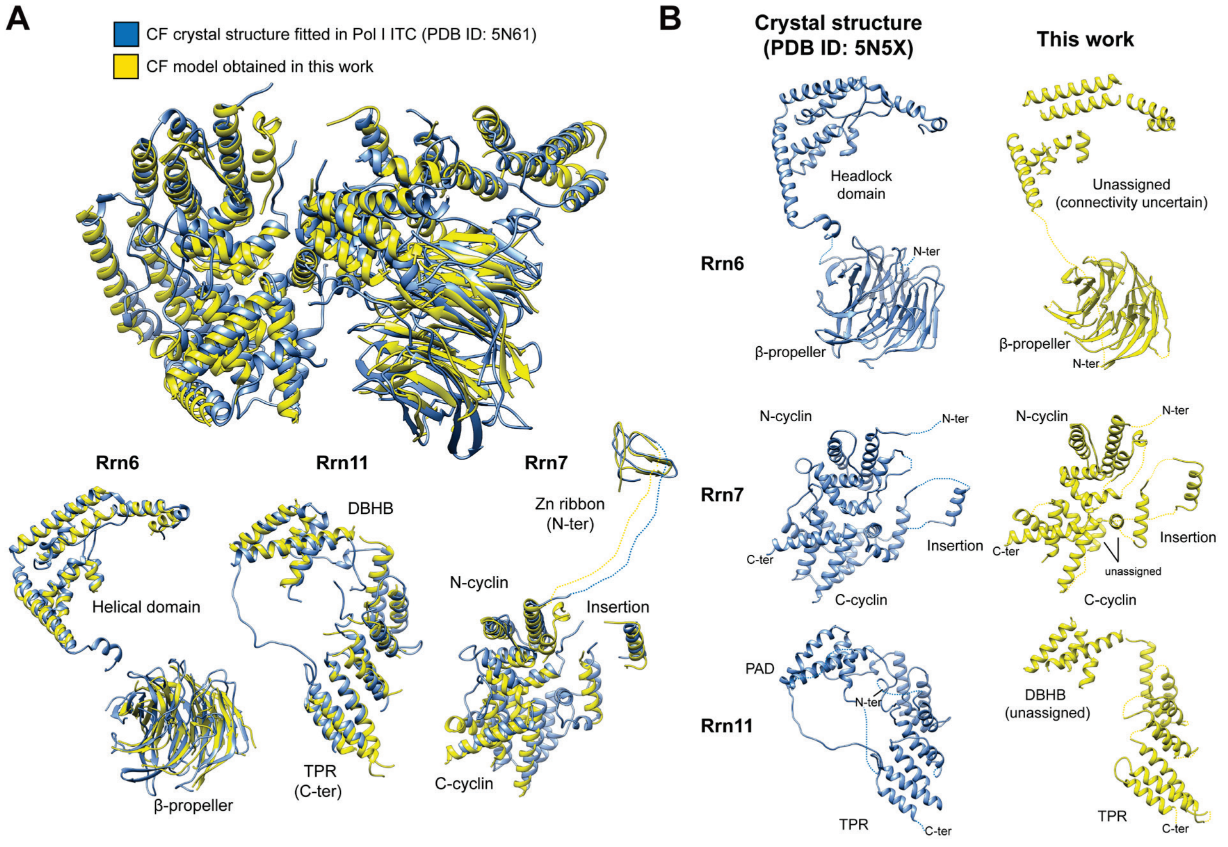

Appendix Figure S3. Comparison of the crystal structure of CF and the CF model obtained in this work. A. Comparison of our model obtained by using a combination of crosslinking-MS, homology modeling and fitting into the EM density (yellow) and the crystal structure fitted into the Pol I ITC (PDB ID 5n61) (light blue) for the full CF structure (top) and individual subunits (bottom). **B.** Comparison of individual subunits between the crystal structure (left, blue) and the model described in this study (right, yellow).

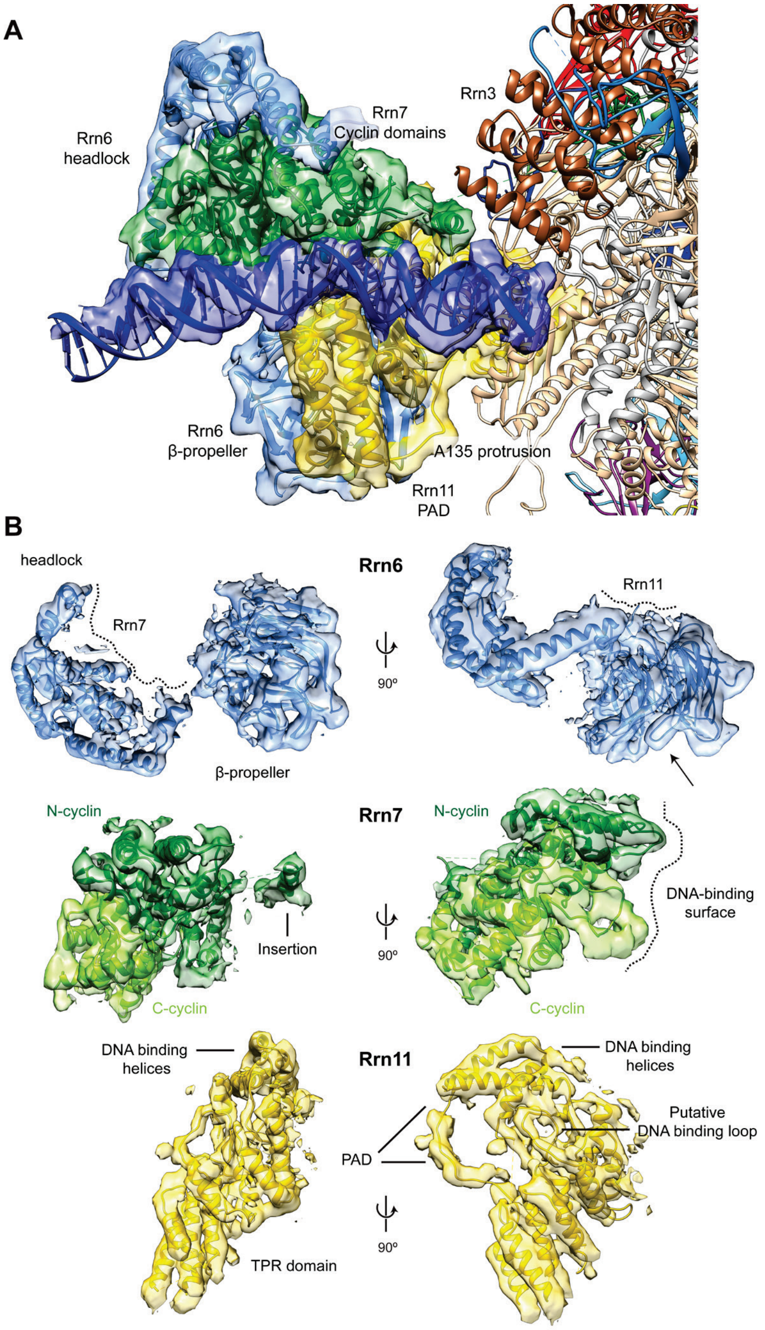

Appendix Figure S4. Crystal structure of Core Factor fitted into the Pol I PIC density. A. Fitting of the CF crystal structure from the ITC accounts for almost the entire CF density in our cryo-EM map. Initially, CF was fitted to the map based on its position in the ITC as a rigid body, and then subunits were fitted individually. The previously unassigned peripheral helices in our model correspond to the headlock domain from Rrn6 and the DBHB corresponds to the N-terminal region of Rrn11. **B.** Extracted density around individual CF subunits. The arrow for Rrn6 indicates the good fit of the β-propeller. Surfaces that lie proximal to Rrn7 and Rrn11 are shown for Rrn6, while the DNA binding surface is shown for Rrn7. For Rrn11, we observe additional density that presumably corresponds to a DNA-interacting loop (residues 282-299) disordered in the crystal structure.
