# Supplementary material for: Structural insights into transcription initiation by yeast RNA polymerase I
Source: EMBO J. 2017 Jul 24;36(18):2698–709. doi: 10.15252/embj.201796958 (PMC5599796; doi:10.15252/embj.201796958)
Supplement: Supplementary file 2 — Expanded View Figures PDF [file EMBJ-36-2698-s002.pdf]

Expanded View Figures

Figure EV1. Cryo-EM of the Pol I PIC.

A FSC curves for the global and focused refinements. The red line indicates the FSC cutoff at 0.143.
B Angular distribution of particles in the final refinement.
C Local resolution mapped onto cryo-EM densities of the globally refined Pol I PIC (upper panel) and focused-refined of CF and Pol I-Rrn3 (in the lower panel, a slice of the same view for each map is also shown).
D The Pol I-Rrn3 focused refinement increases the resolvability of the cryo-EM map. Densities for subunit Rpb8 and for the A190 funnel helices are shown for the global (left) and focused (right) locally filtered maps.
E The density for Rrn3 is presented at a lower threshold. The Pol I-Rrn3 focused refinement map depicted is filtered according to local resolution.

© 2017 The Authors

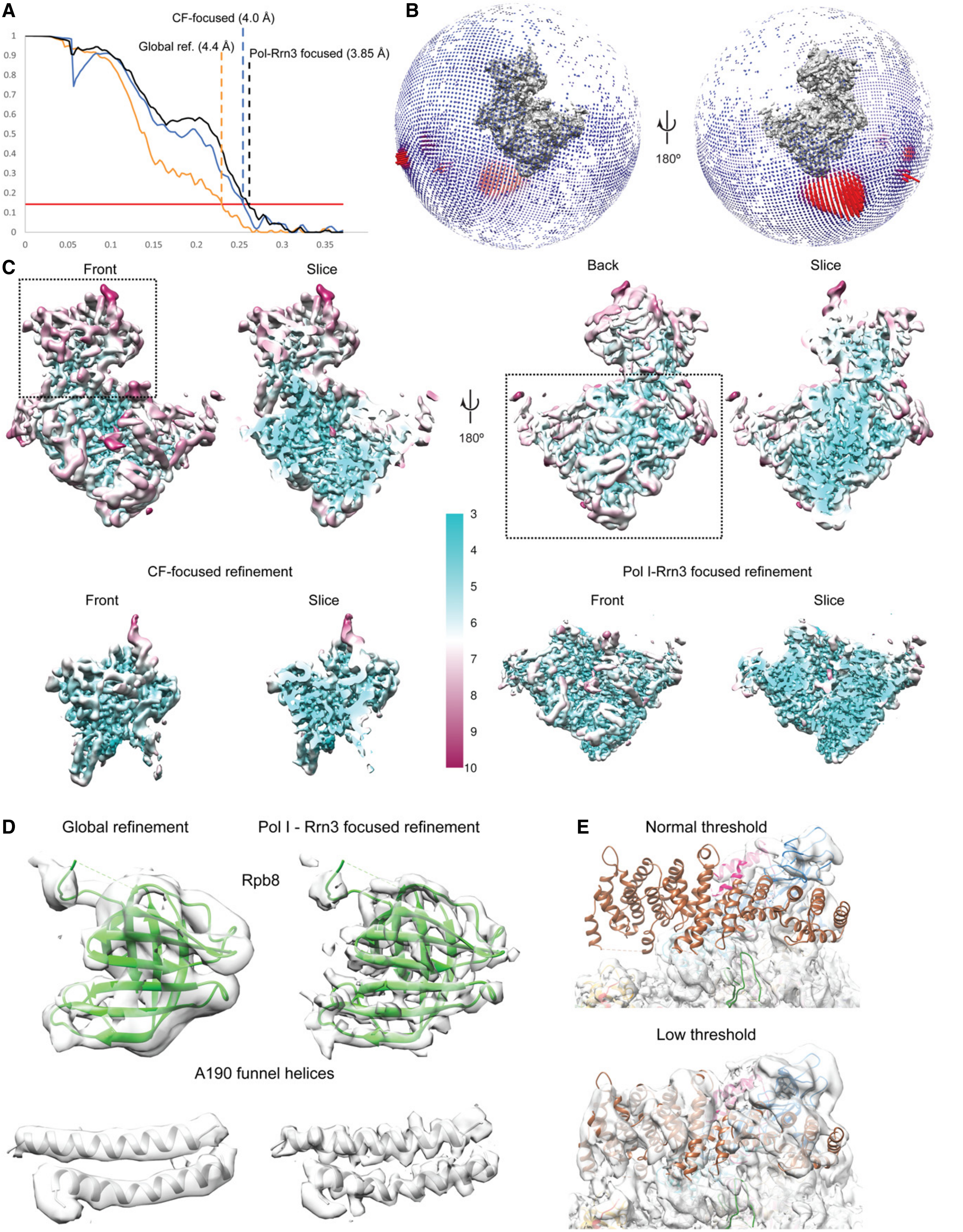

Figure EV1.

 © 2017 The Authors

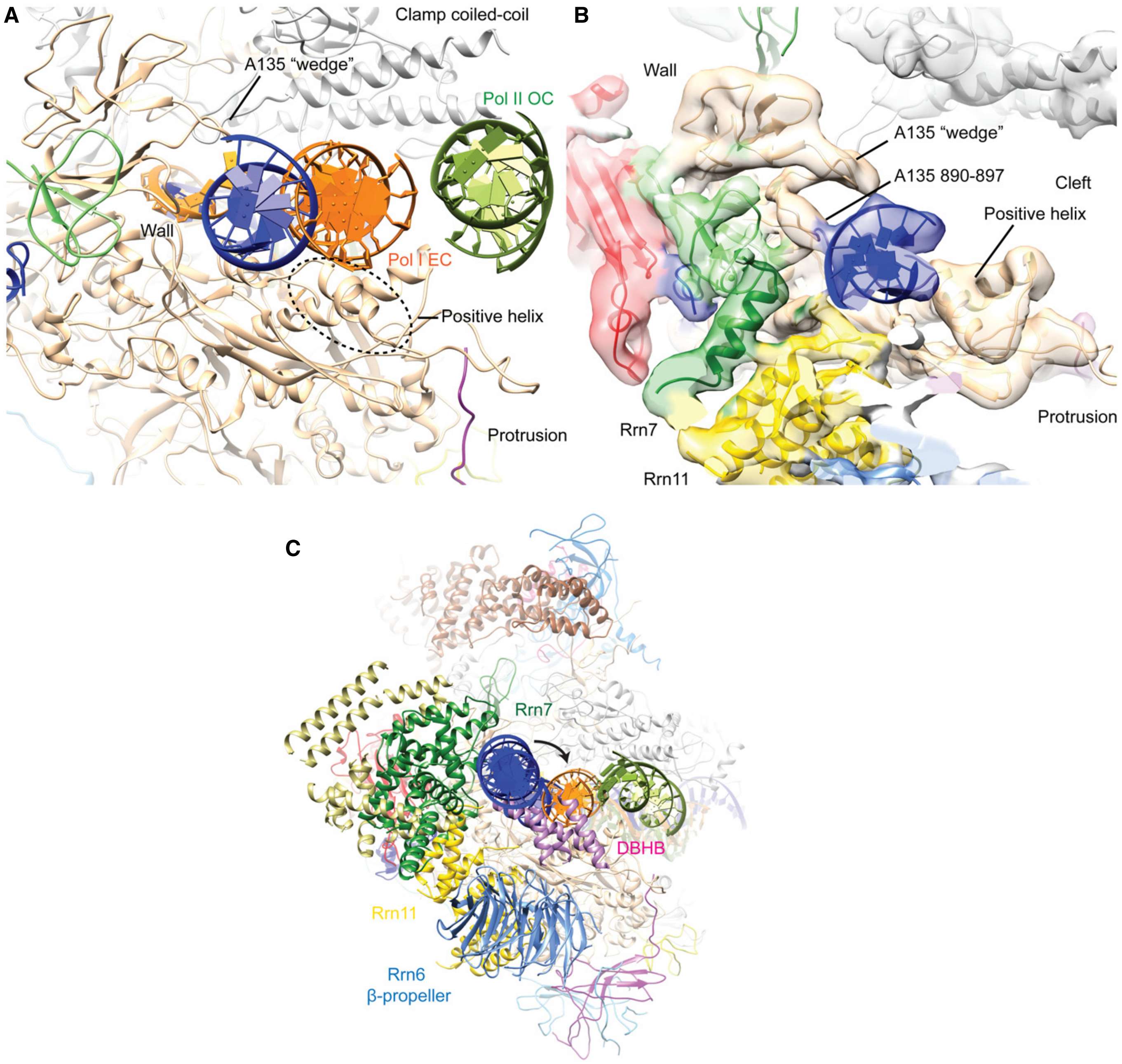

Figure EV2. Upstream DNA position in Pol I PIC.

A The position of the Pol I PIC upstream DNA (blue) is closer to the wall and to the A135 wedge (residues 813–819) compared to the Pol I elongation complex (orange), in which the upstream DNA is closer to the positive helix. In contrast, the upstream DNA for the Pol II OC (dark green, PDB ID 5fyw) is outside the cleft, bound by several Pol II transcription factors (not shown).

B Close-up view on the upstream DNA of the Pol I PIC as in (A). The density of the PIC global refinement map filtered according to local resolution is shown.

C The transition from initiation to elongation in Pol I involves only a small movement of the upstream DNA, indicated by the arrow. In the elongating position, the DNA overlaps with the DBHB.

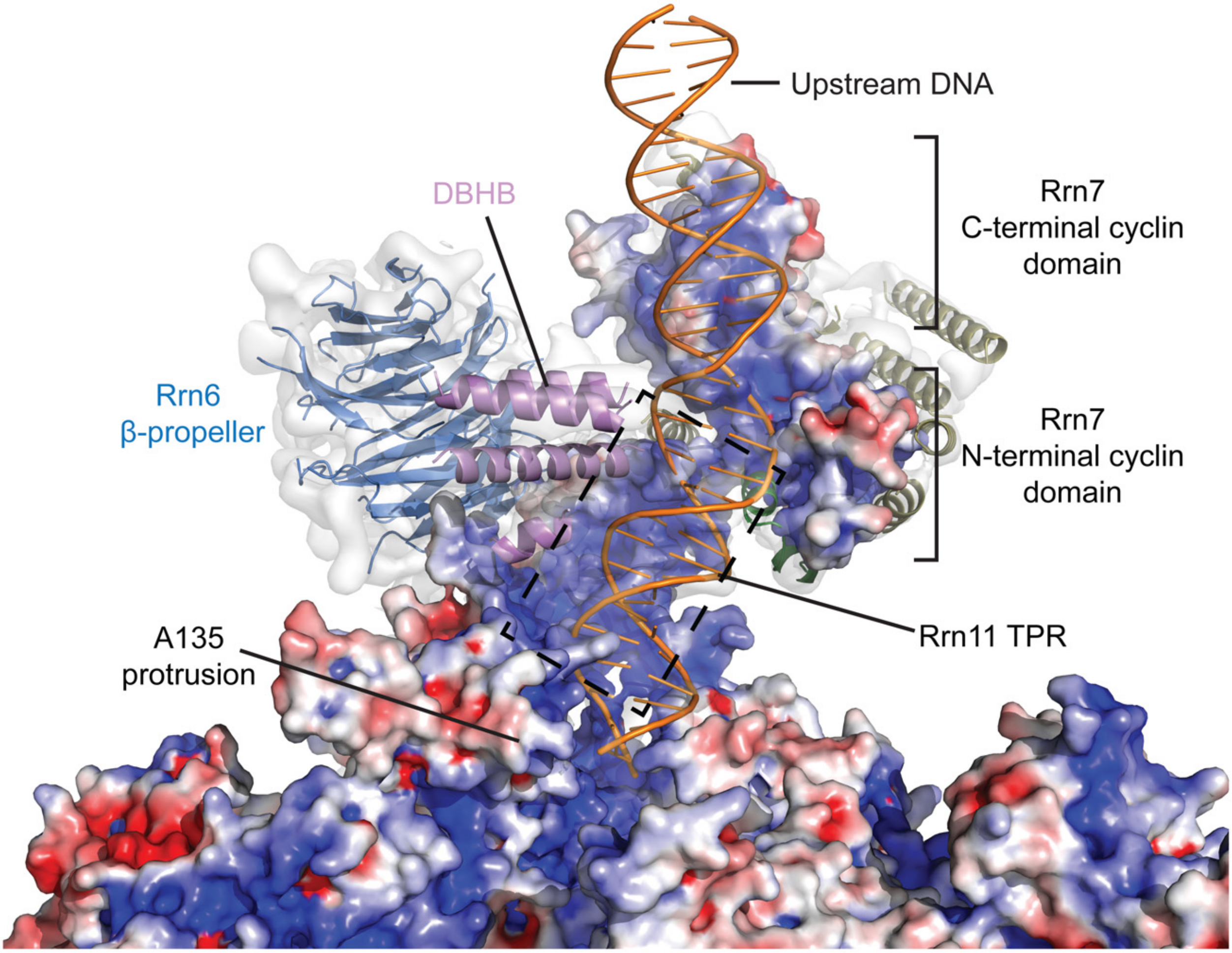

Figure EV3. Electrostatic potential mapped onto the molecular surface of the model.

The positive potential (+5 kT/e) is colored blue, and the negative (−5 kT/e), red. The β-propeller of Rrn6 (blue ribbons) was not used for calculating the charge distribution because its orientation is uncertain. Unassigned helices or regions with uncertain sequence register are depicted as ribbons and colored as in Fig 1.

Figure EV4. Validation of the model by cross-linking and identification of macromolecular folds guided by the cryo-EM density.

▶

A Lysine–lysine cross-links of Pol I PIC obtained in this work. CF subunits are shown as rectangular bars and Pol I subunits as ovals, for the sake of clarity. Inter-links are shown as lines connecting the protein bars, while intra-links are shown as curves. Inter-links between CF subunits are colored red, while inter-links between CF subunits and the Pol subunits are colored according to the color of the respective CF subunit or black for Rrn11. The remaining inter-links are colored black. The domains of CF are indicated. All displayed cross-links have LD (linear-discriminant) confidence score at least 23, as calculated by xQuest (Leitner *et al*, 2014). The figure was created with xiNET (Combe *et al*, 2015).

B Cross-links from CF subunits to Pol I subunits that could be mapped to assigned regions of the model. Cross-links are shown as magenta sticks, and the cross-linked residues are labeled. All cross-link distances are below 30 Å. Several cross-links, in particular those from the C-terminus of Rrn6 and the N-terminus of Rrn11 to various regions of the Pol, cannot be accounted by our model because the corresponding regions of Rrn6 and Rrn11 could not be assigned. At this point, it is unclear whether these cross-links stem from rigid physical interactions of rare oligomers, flexible domains, or false-positive peptide assignments.

C Structures of TPR domains retrieved from the PDB fitted to the locally filtered, CF-focused refinement EM map and compared to the model of Rrn11-TPR built in this work. The TPR structures from the PDB fit the density and validate the fold assignment of the TPR domain of Rrn11. The EM density corresponding to the modeled TPR density is colored yellow. The fits (except τ131, which has not been present in the databases) were obtained by fitting of all TPR domains from CATH (Orengo *et al*, 1997) and SCOP (Murzin *et al*, 1995) databases using PowerFit (van Zundert *et al*, 2016). τ131, which is a TPR domain containing protein from transcription factor IIIC of Pol III, was fitted using Chimera to assess its similarity to Rrn11. Although τ131-TPR has similar curvature as Rrn11, the fit to the density is less optimal than for TPR domains of other proteins. The PDB IDs of the corresponding domains are indicated.

D Structures of β-propeller domains retrieved from the PDB fitted to the EM map. The best fitting structure (PDB ID 1gxr) was used as a template for homology modeling of Rrn6. The fits were obtained by fitting of all β-propeller domains from CATH and SCOP databases using PowerFit.

Source data are available online for this figure.

 © 2017 The Authors

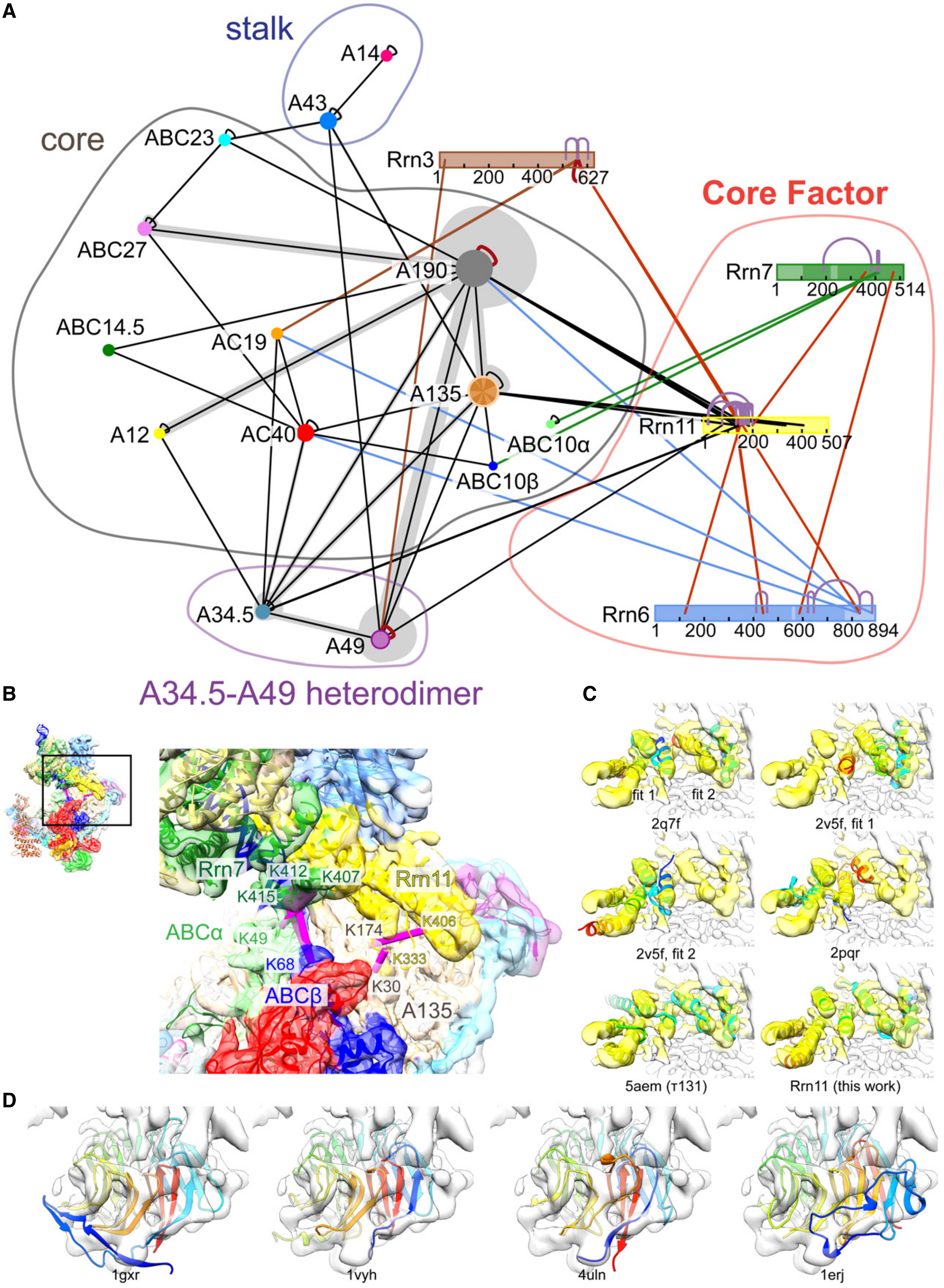
A
stalk
A14
A43
core
ABC23
Rrn3
1 200 400 627
Core Factor
ABC27
A190
Rrn7
1 200 400 514
ABC14.5
AC19
A135
A12
AC40
ABC10α
Rrn11
1 200 400 507
ABC10β
A34.5
A49
Rrn6
1 200 400 600 800 894
A34.5-A49 heterodimer
B
Rrn7
K412
K407
Rrn11
K415
K406
ABCα
K49
K174
K333
K68
K30
ABCβ
A135
C
fit 1
fit 2
2q7f
2v5f, fit 1
2v5f, fit 2
2pqr
5aem (τ131)
Rrn11 (this work)
D
1gxr
1vyh
4uln
1erj

Figure EV4.

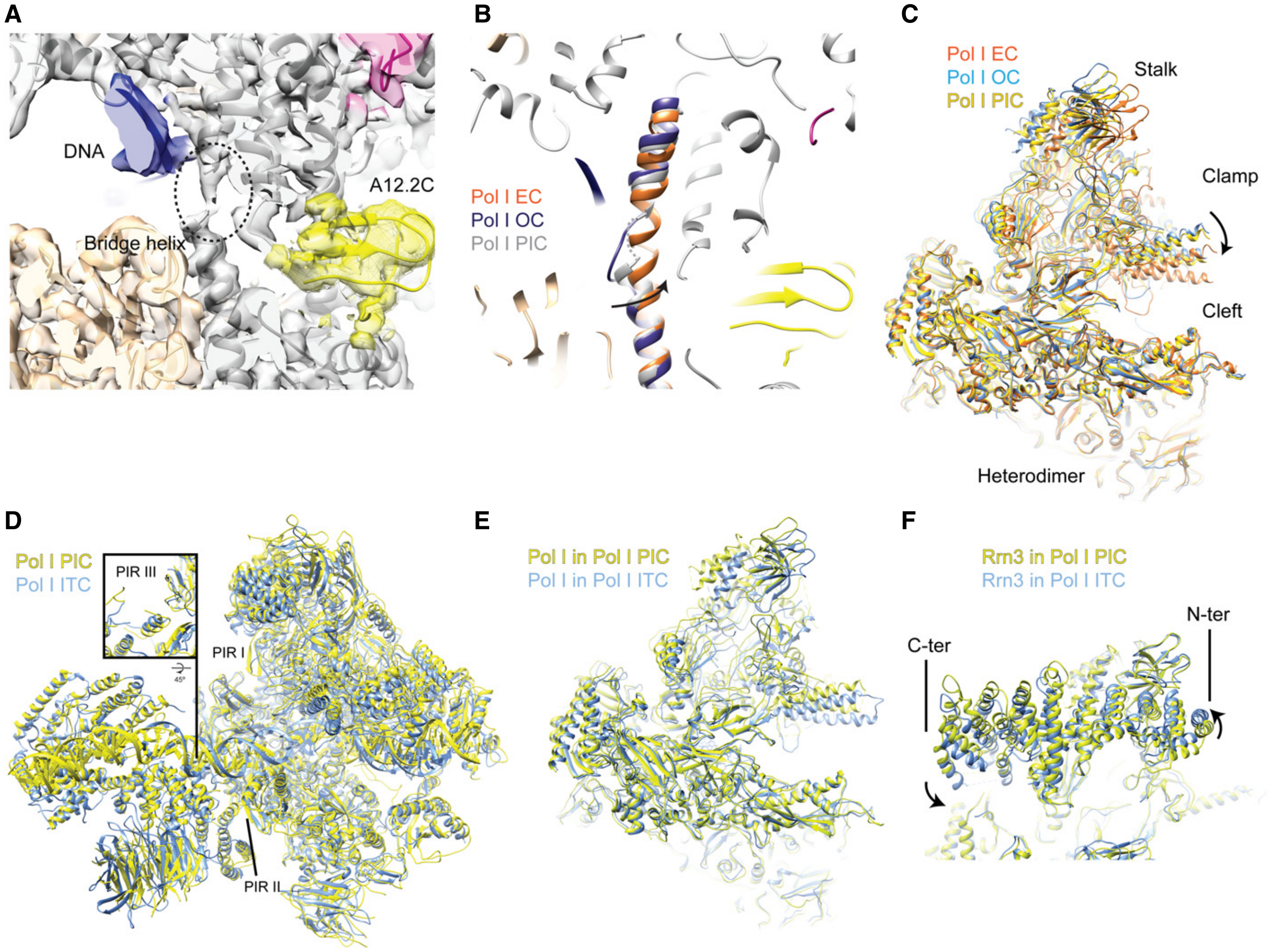

Figure EV5. Pol I conformation in the Pol I PIC.

A The active site of Pol I shows an almost folded bridge helix and the presence of the A12.2 C-terminal domain. Density for the Pol I-Rrn3 focused refinement, filtered according to local resolution, is shown. The density for the A12.2 C is shown as a separate yellow density with a lower threshold.

B Comparison of the bridge helix conformation in the Pol I EC (orange, PDB ID 5m5x), Pol I OC (dark purple, PDB ID 5m5w), and Pol I PIC (gray).

C Comparison of the position of the clamp and cleft closure between the Pol I EC (orange, PDB ID 5m5x), Pol I OC (light blue, PDB ID 5m5w), and Pol I PIC (gold) shows that its conformation is similar to the OC. Models have been superimposed onto subunit A135 in (C–E).

D Comparison of the overall architecture of ITC (blue, Engel *et al*, 2017; PDB ID 5n61) and our model of the Pol I PIC (yellow). Polymerase-interacting regions (PIR) as introduced by Engel *et al* (2017) are indicated illustrating the similarity between both models.

E The cleft is more closed in the ITC (blue, PDB ID 5n61) than in the PIC (yellow).

F Comparison of the position of Rrn3 between the PIC (yellow) and the ITC (blue, PDB ID 5n61) with the models superimposed on subunit A190.

 © 2017 The Authors
