# Supplementary material for: Structural insights into transcription initiation by yeast RNA polymerase I
Source: EMBO J. 2017 Jul 24;36(18):2698–709. doi: 10.15252/embj.201796958 (PMC5599796; doi:10.15252/embj.201796958)
Supplement: Supplementary file 3 — Source Data for EV Figures [file EMBJ-36-2698-s003.zip › SourceData1-related_to_Figure_EV4/96958_readme_sourcedatafigev4.pdf]

Source Data 1, related to Extended View Figure 4A List of highly confident unique crosslinks identified in this work with LD score at least 23 (unique_xlinks.csv) and the complete list of all crosslinks (all_xlinks_combined.csv), including those with lower LD score and mono-links (peptides modified by a crosslinker but not linked to a second peptide), and including additional information such as the sequence of crosslinked peptides. All files in CSV format. Note that some of the crosslinks were identified more than once, hence there may be multiple crosslink entries for a given residue pair in the all_xlinks_combined.csv file.
